# Epigenomic signatures associated with spontaneous and replication stress-induced DNA double strand breaks

**DOI:** 10.3389/fgene.2022.907547

**Published:** 2022-11-24

**Authors:** Sravan Kodali, Silvia Meyer-Nava, Stephen Landry, Arijita Chakraborty, Juan Carlos Rivera-Mulia, Wenyi Feng

**Affiliations:** ^1^ Department of Biochemistry and Molecular Biology, Upstate Medical University, Syracuse, NY, United States; ^2^ Department of Biochemistry, Molecular Biology and Biophysics, University of Minnesota, Minneapolis, MN, United States

**Keywords:** DNA double strand break (DSB), common fragile site (CFS), DNA replication stress, histone H3K36 trimethylation, histone H3K27 trimethylation, CTCF, topologically associated domain (TAD)

## Abstract

Common fragile sites (CFSs) are specific regions of all individuals’ genome that are predisposed to DNA double strand breaks (DSBs) and undergo subsequent rearrangements. CFS formation can be induced *in vitro* by mild level of DNA replication stress, such as DNA polymerase inhibition or nucleotide pool disturbance. The mechanisms of CFS formation have been linked to DNA replication timing control, transcription activities, as well as chromatin organization. However, it is unclear what specific cis- or trans-factors regulate the interplay between replication and transcription that determine CFS formation. We recently reported genome-wide mapping of DNA DSBs under replication stress induced by aphidicolin in human lymphoblastoids for the first time. Here, we systematically compared these DSBs with regards to nearby epigenomic features mapped in the same cell line from published studies. We demonstrate that aphidicolin-induced DSBs are strongly correlated with histone 3 lysine 36 trimethylation, a marker for active transcription. We further demonstrate that this DSB signature is a composite effect by the dual treatment of aphidicolin and its solvent, dimethylsulfoxide, the latter of which potently induces transcription on its own. We also present complementing evidence for the association between DSBs and 3D chromosome architectural domains with high density gene cluster and active transcription. Additionally, we show that while DSBs were detected at all but one of the fourteen finely mapped CFSs, they were not enriched in the CFS core sequences and rather demarcated the CFS core region. Related to this point, DSB density was not higher in large genes of greater than 300 kb, contrary to reported enrichment of CFS sites at these large genes. Finally, replication timing analyses demonstrate that the CFS core region contain initiation events, suggesting that altered replication dynamics are responsible for CFS formation in relatively higher level of replication stress.

## Introduction

CFSs are genomic regions that are prone to DNA strand breakage, observable as gaps or other abnormalities on the metaphase chromosomes. The manifestation, or expression, of CFSs is induced by mild level of DNA replication stress such as DNA polymerase inhibition or nucleotide pool limitation, as reviewed in (Feng and Chakraborty 2017). There are two major mechanisms proposed to underlie CFS formation: defective DNA initiation/progression and replication-transcription conflict (Le Tallec et al., 2014; Ozeri-Galai et al., 2014; Sarni and Kerem 2016). These theories are predicated on the observations that 1) with noted exceptions (El Achkar et al., 2005; Barlow et al., 2013; Handt et al., 2014), CFSs are generally characterized by late replication timing in an unperturbed S phase and experience persistent delay under replication stress (Le Beau et al., 1998; Wang et al., 1999; Hellman et al., 2000; Palakodeti et al., 2004; Pelliccia et al., 2008; Handt et al., 2014); and 2) CFSs tend to nest in large transcribed genes. It is thought that the persistently under-replicated regions, presumably as a result of replication fork breakdown, become unstable and induce genomic rearrangements. Additionally, it is thought that transcription suppresses initiation of DNA replication within these genes, thus contributing to the persistent replication delay (Brison et al., 2019). Finally, direct collisions between the replication and transcription machineries, particularly at sequence locations prone to form R-loops, is thought to cause DNA strand breakage at the CFSs (Helmrich et al., 2011).

CFSs are an intrinsic feature of the human genome and are hot spots for large scale amplification, deletion, and rearrangements, which are thought to underlie genome instability that are prevalent in cancer as well as neurological disorders (Arlt et al., 2003; Glover et al., 2005; Arlt et al., 2006; Ozeri-Galai et al., 2012; Alt et al., 2017; Alt and Schwer 2018; Palumbo and Russo 2019). Therefore, normal cells are arguably the most important model for CFS mapping in order to understand mechanisms of disease onset (Palumbo and Russo 2019). We recently applied the Break-seq method to map DSBs, both spontaneous or chemically induced, in the GM06990 cell line (Chakraborty et al., 2020). These endeavors led to the first high resolution map of DSBs under conditions used to induce CFSs in human lymphoblastoids. The salient points from this study are as follows. First, the vehicle control (DMSO) potently induce DSBs and APH further enhances DSBs; thus APH-induced DSB formation is necessarily a composite effect of the two chemicals. For simplicity we will refer to DSBs induced by both chemicals as APH-induced. Second, both DMSO- and APH-induced DSBs are predominantly located in late-replicating regions, consistent with the noted feature of CFSs. Third, while neither spontaneous nor APH-induced DSBs are enriched for R-loop forming sequences (RLFSs), the DMSO-induced DSBs are enriched for RLFSs, suggesting that transcription induction in the DMSO-treated cells played a major role in DSB formation. Fourth, these DSBs did not show significant correlation with the core sequences of 76 CFSs previously described in lymphoblasts (Le Tallec et al., 2013; Savelyeva and Brueckner 2014). Related to this final point, because CFS cores have been shown to have strong association with large genes of greater than 300 kb (Smith et al., 2006; Le Tallec et al., 2013), our results would suggest that DSBs were not enriched in large genes. Therefore, in this study we investigated the association, or the lack thereof, between DSBs and large genes harboring CFS cores and asked what cis- or trans-factors determine DSB formation.

We systematically examined the relationship between DSBs and select key genomic features mapped by published studies, the majority of which were curated by the Encyclopedia of DNA Elements (ENCODE) project. We specifically focused on data sets generated from the same cell line (GM06990) as our data were, to minimize confounding genetic factors. These data sets included histone modification sites—specifically histone 3 lysine 4 trimethylation (H3K4me3), H3K36me3, and H3K27me3—DNaseI hypersensitive sites (DNaseI HSS), and topologically associated domain (TAD) architectural protein CTCF binding sites. Each of these elements has been implicated in replication and/or transcription regulation (Kimura 2013; Zhang et al., 2015). Of note, it has been suggested that spontaneous DSB sites are correlated with epigenetic markers for chromatin accessibility, including DNaseI HSSs, H3K4me3, and CTCF binding sites (Mourad et al., 2018). We have also found that APH-induced CFSs are associated with TAD boundaries enriched for CTCF binding sites (Sarni et al., 2020). Finally, CTCF binding sites have been shown to be susceptible to DSBs induced by a topoisomerase inhibitor (Canela et al., 2017). Therefore, these studies provided compelling evidence for a connection between DNA strand breakage and 3D genome organization. Additionally, in our previous study (Chakraborty et al., 2020) we analyzed our DSBs for replication timing using Repli-seq data (Hansen et al., 2010); here we also compared the DSB locations to origins of replication mapped by Bubble-seq (Mesner et al., 2013). Our analysis demonstrated a correlation between DMSO- and APH-induced DSBs with H3K36me3 at two locations: first at the TSS, where CTCF bindings sites are also enriched; second within gene bodies downstream from the TSS; origins of replication are broadly distributed at both locales. These observations are consistent with a model where replication stress-induced DSBs are correlated with active transcription and are enriched at TAD boundaries.

## Materials and methods

### Downloaded data sets

DNaseI HSS_1: bigWig

https://www.encodeproject.org/files/ENCFF529JFV/@@download/ENCFF529JFV.bigWig.DNaseIHSS2:bigWig

DNaseI HSS_1: bigWig

https://www.encodeproject.org/files/ENCFF709PEX/@@download/ENCFF709PEX.bigWig 

DNaseIHSS_1: bigBed narrowPeak

https://www.encodeproject.org/files/ENCFF735IVN/@@download/ENCFF735IVN.bigBed 

DNaseIHSS_2: bigBed narrowPeak

https://www.encodeproject.org/files/ENCFF043DMN/@@download/ENCFF043DMN.bigBed 

CTCF ChIP-seq: bigWig

https://www.encodeproject.org/files/ENCFF469OOI/@@download/ENCFF469OOI.bigWig 

CTCF ChIP-seq: bed narrowPeak

https://www.encodeproject.org/files/ENCFF276JDQ/@@download/ENCFF276JDQ.bed.gz 

H3K4me3 ChIP-seq: bigWig

https://www.encodeproject.org/files/ENCFF965GIX/@@download/ENCFF965GIX.bigWig 

H3K4me3 ChIP-seq: bed narrowPeak

https://www.encodeproject.org/files/ENCFF357ALO/@@download/ENCFF357ALO.bed.gz 

H3K27me3 ChIP-seq: bigWig

https://www.encodeproject.org/files/ENCFF533NLA/@@download/ENCFF533NLA.bigWig 

H3K27me3 ChIP-seq: bed narrowPeak

https://www.encodeproject.org/files/ENCFF554UCC/@@download/ENCFF554UCC.bed.gz 

H3K36me3 ChIP-seq: bigWig

https://www.encodeproject.org/files/ENCFF324YFT/@@download/ENCFF324YFT.bigWig 

H3K36me3 ChIP-seq: bed narrowPeak

https://www.encodeproject.org/files/ENCFF372NOF/@@download/ENCFF372NOF.bed.gz 

Bubble-seq origins of replication data (GSE38809_GM_combined_RD_bubbles.bedgraph) were downloaded from GEO accession number GSE38809.

#### MEME suite searches

The commands used for the following search engines are listed below.

AME: ame--verbose 1 --oc. --scoring avg--method fisher--hit-lo-fraction 0.25 --evalue-report-threshold 10.0 --control--shuffle-- --kmer 2 DSBFILE.fasta db/HUMAN/HOCOMOCOv11_core_HUMAN_mono_meme_format.meme

STREME: streme--verbosity 1 --oc. --dna--totallength 4000000 --time 14400 --minw 8 --maxw 15 --thresh 0.05 --align center--p DSBFILE.fasta

Tomtom: tomtom -no-ssc -oc. -verbosity 1 -min-overlap 5 -mi 1 -dist pearson -evalue -thresh 10.0 -time 300 query_motifs db/HUMAN/HOCOMOCOv11_core_HUMAN_mono_meme_format.meme

#### Random simulation test for correlation between DSBs and chromatin features

The association between a DSB and a chromatin feature was determined by whether the two regions overlap by at least 1 bp using intersectBed function in BEDTools. Random DSB sequences were generated by shuffleBed with the same number of DSBs per chromosome. The shuffled DSBs were then compared to the chromatin feature for overlaps. The random simulation was performed 1000 times in each test. The number of simulations in which the shuffled DSBs overlapped with the chromatin feature at an equal or higher frequency than the real data was divided by the number of simulations to calculate the *p* value.

#### GP-seq score calculation for DSBs

GP-seq scores were downloaded from the GEO database under the accession number GSE135882 for experiment 5 (GSE135882_Exp5.1 Mb). GP-seq scores in each unique DSB region were tallied, averaged, and plotted.

#### Random forest prediction of classifier for DSB formation

We used the R package “randomForest” to build and train the random forest models employed in this study. We left mtry on default and used a value of 700 for ntree which represents the number of decision trees used by the model. We set importance to TRUE in order to access the MeanDecreaseAccuracy and MeanDecreaseGini values as well as the variable importance plots. The ‘pROC’ package was installed to plot the ROC (receiver operating characteristic curve) and PR (Precision Recall) curves. We used the BigWigAverageOverBed function to calculate the feature signals over DSB regions and used the mean values as input variables. Random sampling of DSBs from the genome was accomplished using the shuffleBed function from the BEDTools suite.

#### Repli-seq

At least 2 × 10^6^ cells were used for each Repli-seq experiment. Cells were treated with DMSO, or 0.3 µM, or 0.6 µM APH, or nothing at all for 24 h. BrdU was then added at 100 µM and cells were incubated for 2 h before washing with PBS and harvesting, followed by ethanol fixation. Fixed cells were then sorted by flow cytometry into early and late-replicating fractions. BrdU-labeled DNA from each fraction was immunoprecipitated, followed by preparation for sequencing libraries as previously described (Rivera-Mulia et al., 2022). Two biological replicates were produced for each sample, with similar results. Replication timing profiles from one replicate are shown.

## Results

### APH-induced DSBs are a composite of those induced by replication stress through APH and transcriptional upregulation by DMSO

**FIGURE 1:**
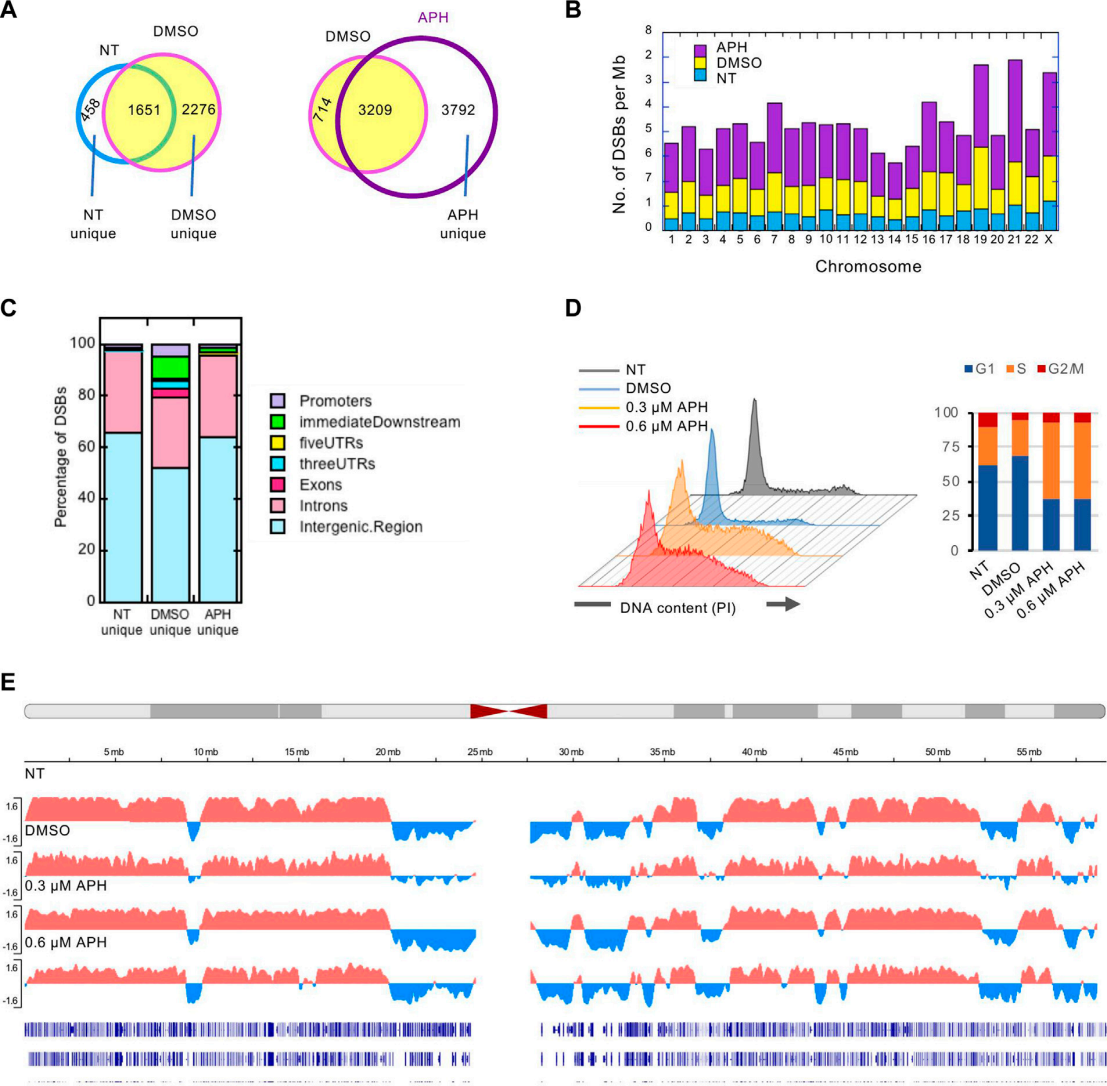
Break-seq mapping of DSBs in normal human lymphoblastoids. **(A)** Concordance between DSBs in pair-wise comparisons and subsetting DSBs unique to each condition. **(B)** DSB density (per Mb of DNA) across each chromosome. **(C)** Distribution of DSBs unique to each condition with respect to genes. **(D)** Cell cycle analysis of cells with the indicated treatment by flow cytometry. PI: propidium iodide. **(E)** Replication timing analysis under replication stress. Representative replication timing profile plotting the RT Log2 Ratio for chr19 is shown for each treatment. Bottom tracks represent RefSeq genes.

We recently generated high resolution mapping of genome-wide DSB sites in human lymphoblastoid (GM06990) cells (Chakraborty et al., 2020). We used conditions known to induce CFS formation, i.e., mild level of replication stress by APH, with equal volume of the vehicle, dimethyl sulfoxide (DMSO), or no treatment (NT) at all as controls. This study produced 2111 NT or spontaneous DSBs, and 3927 and 7002 DSBs in DMSO- and APH-treated cells, respectively, demonstrating drug-specific induction of DSBs (Figure 1A). Spontaneous DSBs did not show apparent chromosomal bias; in contrast, DMSO-treated cells showed an enrichment of DSBs on chr19 whereas APH-treated cells had the highest density of DSBs on chr21, followed by chr19 (Figure 1B). We compared the DSBs to derive those shared and those uniquely induced by each condition. The vast majority (>78%) of the spontaneous DSBs were also present in the DMSO- and APH-treated cells, suggesting that an integral component of the CFSs are those regions of the genome that are intrinsically susceptible to DSBs (Figure 1A). However, DMSO apparently elicited a strong induction of 2276 DSBs not present in the NT sample (“DMSO-unique”). We have shown that the genic association of DSBs increased from 33 to 41% from untreated cells to DMSO-treated cells (Chakraborty et al., 2020). Here we further showed that ∼50% of the “DMSO-unique” DSBs occur in genic regions (Figure 1C), suggesting that transcription increase in DMSO cells caused DSBs. In contrast, the genic association level dropped to ∼38% for “APH-unique” DSBs, though still a slightly higher level compared to NT-unique DSBs (Figure 1C), suggesting that transcription repression by APH-induced replication stress. Thus, we concluded that DSBs in APH samples are a composite effect of transcription induction by DMSO and repression by APH.

Previously, we have shown that DMSO- and APH-induced DSBs, but not spontaneous DSBs, are significantly enriched in late-replicating domains, using published Repli-Seq data for the GM06990 cell line (Hansen et al., 2010) to establish autosomal early- and late-replicating domains (Chakraborty et al., 2020). These observations are consistent with the known characteristics of the CFSs. To test if the same DSB-associated late-replicating regions remain late-replicating in DMSO or APH treatment, we performed Repli-seq experiments (Rivera-Mulia et al., 2022) with cells treated with DMSO, 0.3 μM or 0.6 μM APH, or nothing at all. Cells were transiently (2 h) labeled by BrdU, followed by sorting into early and late-replicating fractions by flow cytometry. BrdU-labeled nascent DNA was then immunoprecipitated from both fractions and subjected to next-generation sequencing, producing replication timing profiles represented as the Log2 ratios of sequence reads of early *vs*. late fraction. The result showed that 0.3 µM APH, the dosage at which the majority of our Break-seq experiments were conducted, caused S-phase arrest compared to the NT and DMSO controls (Figure 1D). Treatment with 0.6 µM APH also induced S-phase arrest (Figure 1D). Nevertheless, genome-wide replication timing profiles demonstrated that most of the normallly late-replicating regions remain late-replicating in drug-treated samples (Figure 1E), with few exceptions in the 0.6 µM APH treatment that produced some local advanced replication timing among a large late-replicating region (more later). These results are consistent with our previous findings of global preservation of replication timing after APH treatment in other cell types (Sarni et al., 2020). We next asked how these DSBs are distributed in the CFSs.

### APH-induced DSBs demarcate CFS core sequences and are not enriched in large genes

**FIGURE 2:**
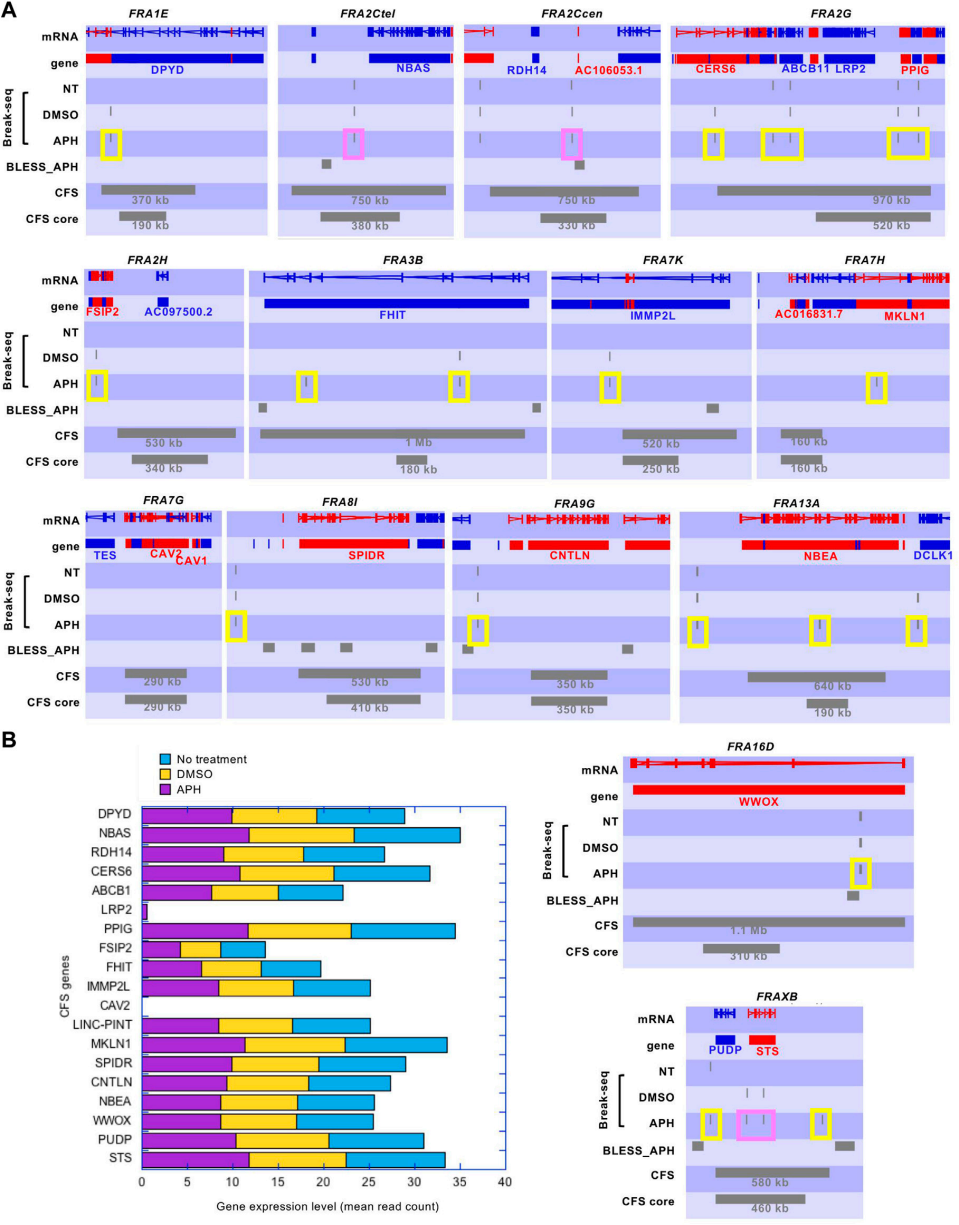
Break-seq signals tend to demarcate the boundaries of molecularly characterized CFSs. **(A)** SeqMonk profiles for DSB tracks. Genes encoded on the + strand and–strand are represented by red and blue blocks, respectively. DSB signals and CFS or CFS core regions are represented by grey blocks. Break-seq samples of NT (not treated)-, DMSO-, or APH-treated normal human lymphoblastoids (GM06990) are shown. The published APH-induced DSBs by BLESS are also shown. Note that Break-seq is more sensitive and have higher resolution than BLESS. The size of CFS and CFS core regions are shown. Those Break-seq signals flanking the CFS or CFS core regions are labeled by a yellow box, and those located within the CFS or CFS core regions are labeled by a pink box. **(B)** Expression levels of genes at CFSs measured by RNA-seq in cells treated with 0.3 µM APH, or DMSO, or nothing at all, for 24 h. Detailed RNA-seq data analysis and raw data are described elsewhere (Chakraborty et al., 2020. in press) and accessible from the GEO accession number GSE124403.

It was estimated that approximately one-third of CFSs are associated with large genes (Smith et al., 2006). A recent study systematically defined these long (>300 kb) transcribed genes which experience significantly delayed replication in APH and showed that they correspond to CFS core regions reported in the literature (Brison et al., 2019). These CFS core regions were defined by higher resolution FISH (fluorescence *in situ* hybridization) probes in fourteen molecularly characterized CFSs (Savelyeva and Brueckner 2014). We first compared the DSBs in our Break-Seq experiments to these large transcribed genes with delayed replication (henceforth"large genes”), and found no significant correlation (*p* < 0.001) (Chakraborty et al., 2020). Moreover, the APH-induced DSBs mapped by another sequencing-based method called BLESS (Crosetto et al., 2013) did not show correlation with large genes either, while they showed significant correlation with DSBs mapped in our study (*p* < 0.001) (Chakraborty et al., 2020). We then systematicaly examined the fourteen molecularly characterized CFSs and we observed only 1 to 2 discrete spots of APH-induced DSBs in all of these CFS regions, even in large genes (*e.g., FHIT* and *WWOX,* sized 1502 and 1113 kb, respectively) associated with FRA3B and FRA16D, respectively (Figure 2A, “Break-seq” tracks). This relatively low density of DSBs in CFSs was also observed for the BLESS data set (Figure 2A, “BLESS_APH” track). Importantly, transcriptomic analysis showed that these CFS-associated genes were expressed at similar levels in all conditions (Figure 2B), with the exception of *CAV2* at *FRA7G*, where we did not detect expression, nor any DSB, in APH (Figures 2A,B). *LRP2* at *FRA2G* showed only moderate expression in APH and not in NT or DMSO samples, and the DSBs at the *FRA2G* locus were detected elsewhere with expressed genes (Figures 2A,B). Thus, despite a genome-wide transcription repression by APH, CFS-associated genes remain active.

**FIGURE 3:**
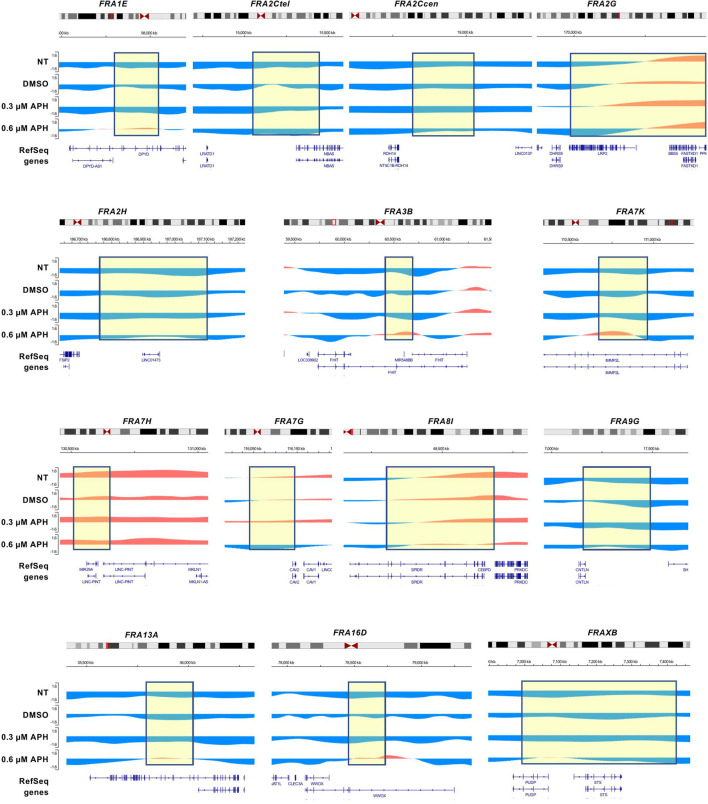
CFS core regions advance their replication timing under replication stress. Replication timing profiles at CFS regions under distinct treatment with APH. The boxed regions correspond to CFS core sequences shown in Figure 2. The positions of the CFS core are approximate.

**FIGURE 4:**
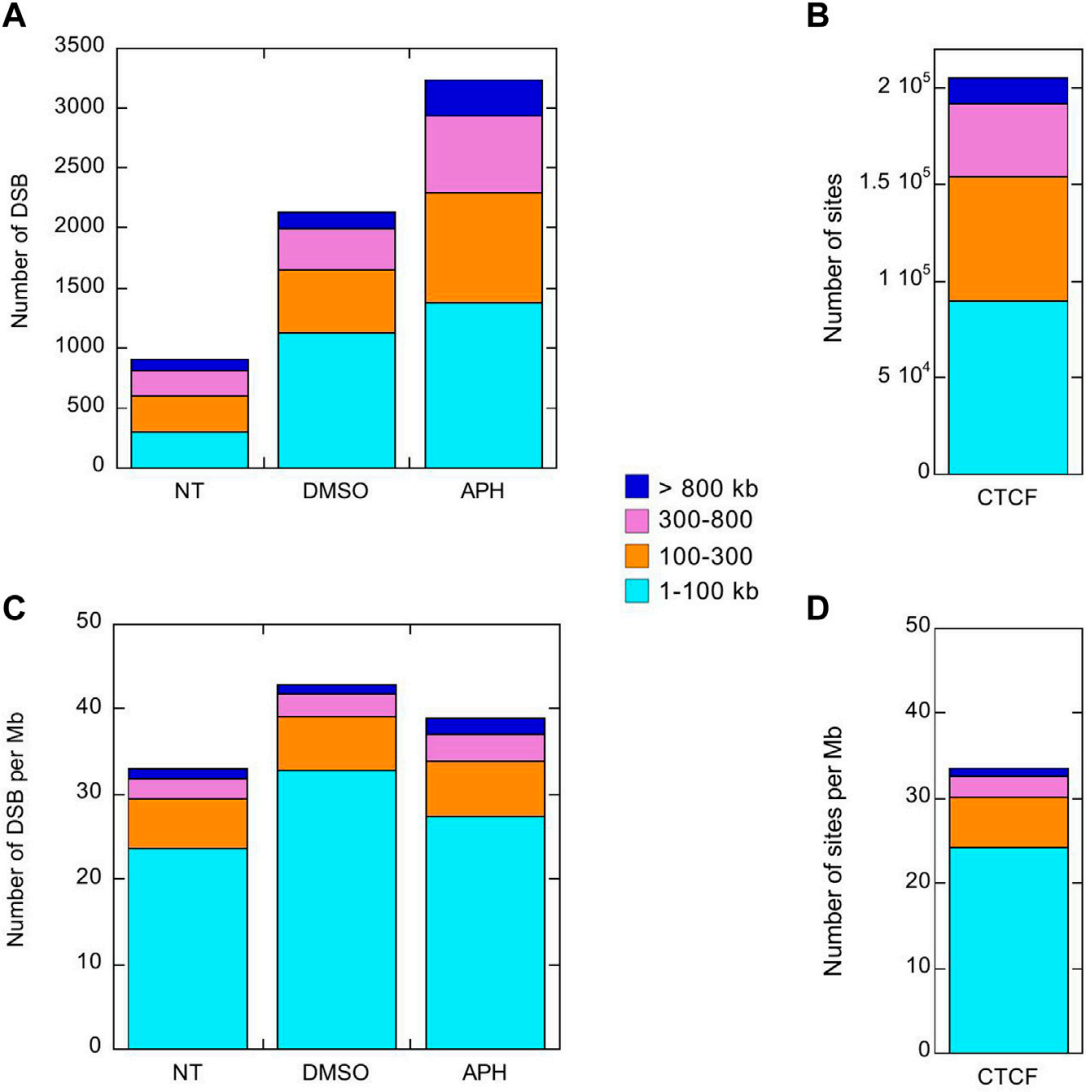
DSBs induced by DMSO or APH are present but not enriched in large genes >300 kb. Stacked column plots of the number of DSBs **(A)** and CTCF binding sites **(B)** in the indicated gene size groups. Stacked column plots of the density of DSBs **(C)** and CTCF binding sites **(D)**, *i.e.*, number per Mb of DNA, in the indicated gene size groups.

Interestingly, both the Break-seq and BLESS-derived APH-induced DSBs tend to demarcate the boundaries of the mapped CFSs or CFS cores (Figure 2A). The relatively sparse nature of DSBs at these CFSs might be due to the relatively low APH concentrations (0.03 and 0.3 µM) employed by Break-seq experiments. Notably, we observed that when cells were treated with 0.6 µM APH it resulted in local late-to-early replication timing changes at 9 of the 14 CFS core regions, the exceptions being FRA2Ctel, 2G, 7H, 7G, and 8I (Figure 3). FRA2Ctel actually showed further delay in replication timing within the CFS core. Among the CFS cores with advanced timing, six (FRA1E, 3B, 7K, 13A, 16D and XB) clearly demonstrated initiation events within the core (Figure 3). These advanced timing changes suggested that the CFS core regions might contain dormant origins that are activated upon replication stress. They further suggested that altered replication dynamics within the CFS contribute to the DSB formation at higher APH dosages (see discussion). We then proceeded to further investigate the relationship between DSBs and genes in different size groups defined as 1–100, 100–300, 300–800, and >800 kb. Results showed that the number of DSBs as well as DSB density (number of DSBs per Mb of DNA) decreased with increasing gene size (Figures 4A,C). This trend was observed in both spontaneous DSBs and drug-induced ones. Notably, DSB density in all gene groups increased with DMSO/APH treatment compared to “NT” control (Figure 4B). However, it was the gene group of 1–100 kb, the smallest of all, that saw the biggest increase in DMSO-treated cells (from 24 per Mb of DNA in untreated cells to 33 per Mb of DNA), followed by a decrease with APH treatment (down to 27 per Mb of DNA). This result led us to hypothesize that the smaller gene groups, particularly the 1–100 kb group, were physically clustered and the gene cluster underwent DMSO-induced transcription and APH-induced replication fork impediments, which collectively led to the increase in DSB density. Thus, our data strongly suggest an underlying role of genome organization in drug-induced DSB formation.

### CTCF binding site density mirrors that of DSB density across gene groups

A recent study has shown that APH-induced CFSs correspond to TAD boundaries that are significantly delayed in replication timing (Sarni et al., 2020). TAD boundaries are delineated by CCCTC binding factor (CTCF) to form chromatin loops containing sequences with similar transcriptional regulation. We reasoned that large genes would require relatively fewer CTCF binding events for organization. In contrast, a similarly sized gene cluster housing many small genes might be organized into multiple smaller chromatin loops with distinct environment, which would require proportionally more CTCF binding sites. In other words, the density of chromatin loops should correlate with gene density. Indeed, we showed that the number and the density of CTCF binding sites decreased as gene size increased, a pattern that is almost identical to that of DSBs, particularly for those induced by APH (Figures 4C,D). This result suggested that DSB formation is determined by chromatin organization through CTCF binding and related events. Therefore, we sought to further test our hypothesis by investigating the relationship between DSBs and known chromatin accessibility markers.

### Drug-induced DSBs locations are strongly correlated with TSS and the histone marker H3K36me3

**FIGURE 5:**
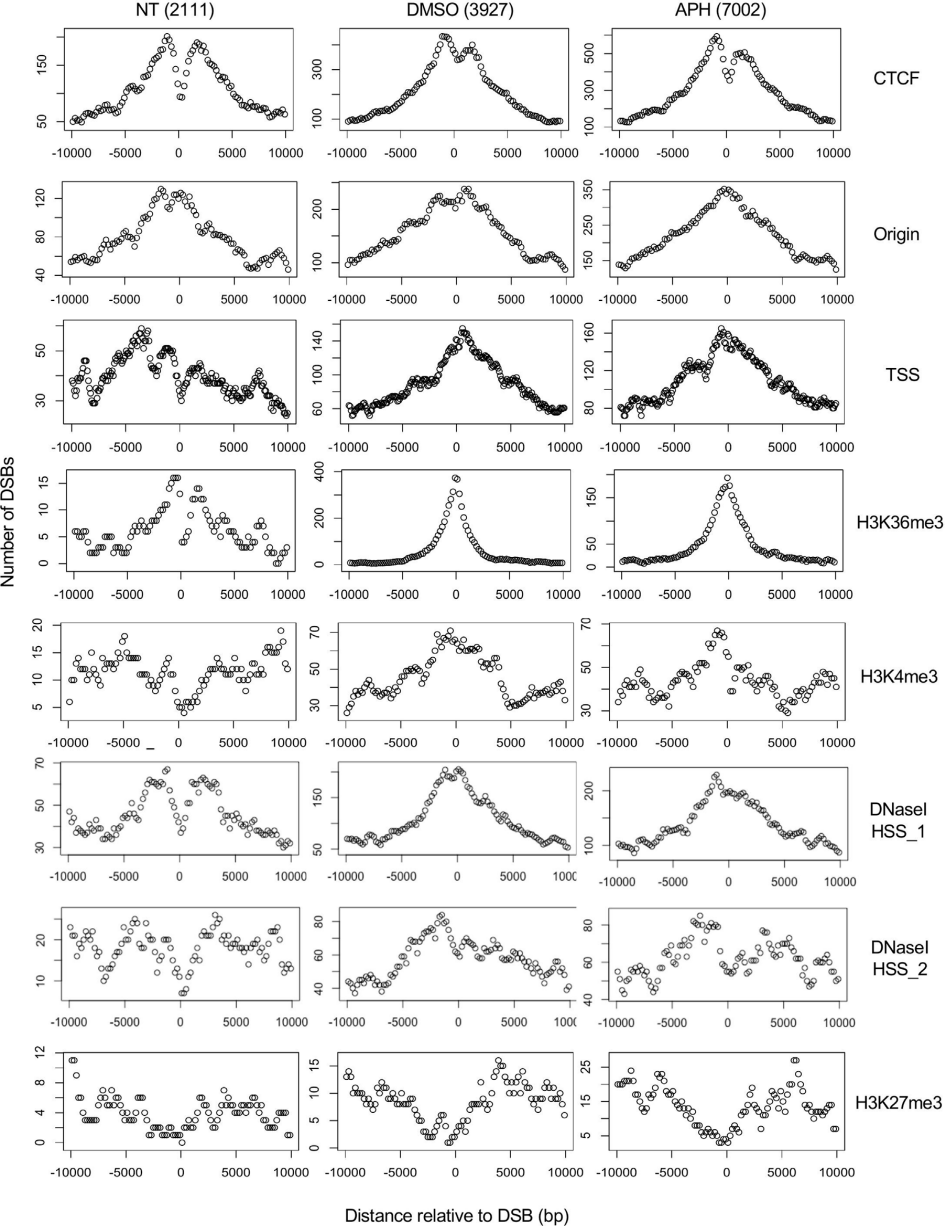
Aggregated plots of DSBs around the nearest chromatin marker. The number of DSBs in each of the 50 bins across a 20,000 bp window centered on the given chromatin marker are scored and plotted on the *Y*-axis against the relative distance to the center of the chromatin marker (*X*-axis). “TSS” and “H3K36me3” are the only two markers that were found associated with DSBs in DMSO- and APH-treated samples at a genomic scale (see Supplementary Figure S2).

We took advantage of the ENCODE project which cataloged a large set of genome-scale experiments of mapping chromatin structures. We specifically focused on those data generated from GM06990, the same cell line used in our Break-seq mapping experiments. We systematically analyzed the distribution of DSBs over each of the chromatin features including TSS, CTCF binding sites, DNaseI HSSs, H3K4me3, H3K27me3, H3K36me3, and origins of replication (Supplementary Figure S1A), whose genomic distributions were summarized in Supplementary Figure S1B. We began by analyzing the distribution of DSBs over a 20-kb window centered on each feature in order to assess if DSBs were likely to associate with any of the features. Note that all these features were mapped in untreated cells, therefore these analyses allowed us to specifically test if and how epigenomic features in otherwise normal cells impact the DSBs seen in DMSO- or APH-treated cells. Spontaneous DSBs showed moderate association with only CTCF binding sites and origins, and no apparent association with the other features (Figure 5). Interestingly, DSBs in DMSO- and APH-treated cells showed an even stronger association with CTCF binding sites and origins (Figure 5, note the different *y*-axis scale on each plot). It is important to note that the association between CTCF binding sites and DSBs was not statistically significant when compared to randomized DSBs, suggesting that the comparison of DSBs to CTCF binding sites alone might not be sufficient to discern the relationship between DSBs and epigenomic features.

We next observed that drug-induced DSBs showed strong correlation with TSS and H3K36me3, moderately with H3K4me3 and DNaseI HSS, but not with H3K27me3, suggesting that DSBs are dependent on active transcription and not merely high chromatin accessibility (Figure 5). Moreover, we demonstrated that the drug-induced DSBs are correlated with TSS and H3K36me3 genome-wide by analyzing the distribution of DSBs overall features in the genome and not just the nearest ones (Supplementary Figure S2A). Specifically, 543 (13.8%) and 970 (13.9%) of the DMSO- and APH-induced DSBs, respectively, overlapped with H3K36me3 sites. These overlaps were significantly higher compared to randomized sequences in a random simulation test with >1000 iterations (*p* < 0.001). Because H3K36me3 is associated with active transcription and is enriched in the gene bodies downstream from TSS (Supplementary Figure S2B), the observed correlation of DSBs with both TSS and H3K36me3 suggested a strong dependence on transcription.

Furthermore, CTCF binding sites are strongly associated with TSSs and a small fraction of CTCF binding sites are also associated with H3K36me3 (Supplementary Figures S2C,D). Therefore, it appears that a subset of CTCF binding sites and a subset of H3K36me3 are associated with each other at the TSS, whereas the remainder of H3K36me3 are distributed downstream of TSS in gene bodies. These results led us to conclude that DSBs are associated with both H3K36me3 sites at the TSS as well as those downstream from TSS, suggesting a transcription-dependent mechanism of DSB formation. Moreover, APH-induced DSBs were better correlated with origins compared to spontaneous or DMSO-induced DSBs (Figure 5), consistent with induced replication stress impacting forks emanating from the origins. Finally, we showed that origins are broadly distributed around H3K36me3, CTCF binding sites, and TSS, in descending order of proximity (Supplementary Figures S3A–C). Therefore, it suggests that DSB formation at both TSS and gene bodies was also regulated by origin activities and replication fork movement.

### Chromosomes enriched for DMSO- or APH-induced DSBs tend to be located in the radial center of the nucleus

**FIGURE 6:**
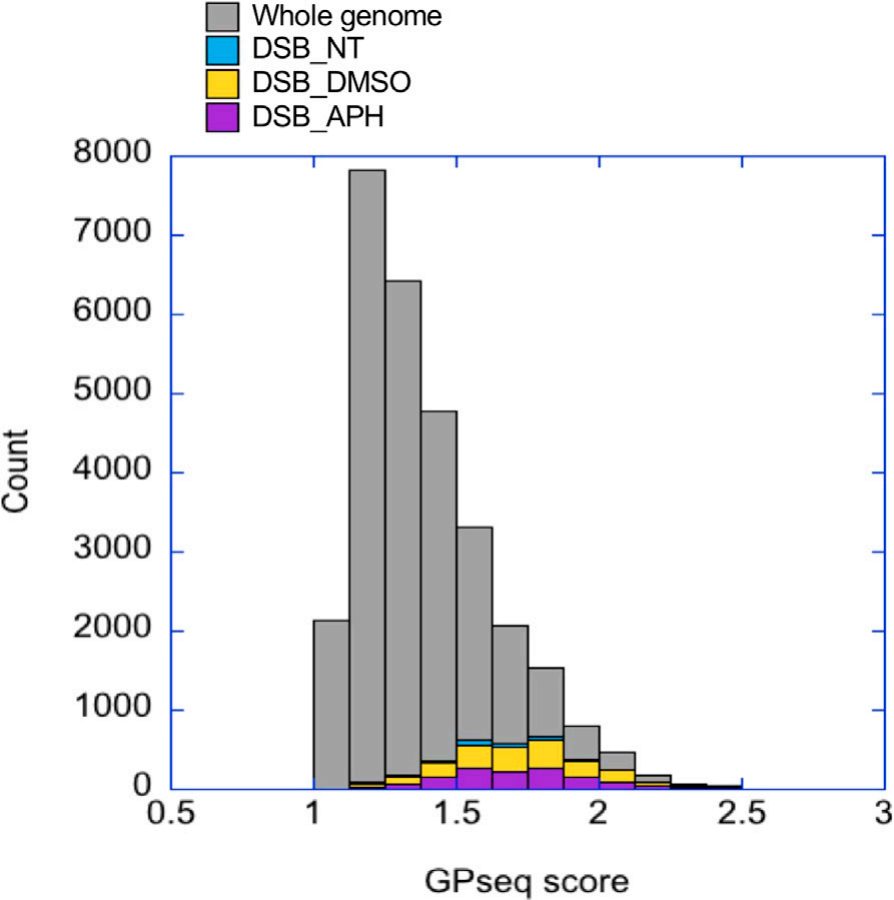
GP-seq scores of DSBs are higher than genomic average. The GP-seq scores for DSBs in each treatment are shown in a stacked column plots as a histogram. The GP-seq scores of the genomic average are also shown as a histogram.

To further discover defining features for these stress-induced DSBs we derived a list of DSBs overlapping with all features including H3K36me3, CTCF binding site, and origin (Data File S1). We found 24, 187, and 138 such DSBs in the untreated, DMSO-, and APH-treated samples, respectively. We first asked if there was any chromosomal bias of the DSBs. Remarkably, the chromosome with the highest enrichment of DSBs in all conditions was Chr19. While spontaneous DSBs were only enriched on Chr19, drug-induced DSBs also showed enrichment on Chr5, 17, and 22 for DMSO and Chr15, 17, and 22 for APH treatment. These chromosomes all tend to be located near the radial center of the nucleus based on an elegant study using Genome Positioning (GP)-seq to analyze the 3D chromosome positioning (Girelli et al., 2020). Among them Chr19 has the highest GP-seq score, *i.e.*, the shortest radial distance from the center of the nucleus (Girelli et al., 2020). Indeed, compared to genomic average distribution of GP-seq score, the DSBs in our study showed skewed distribution towards higher GP-seq scores, hence nearer to the radial center of the nucleus (Figure 6). Girelli et al. further demonstrated that DSB level, using γH2A.X as a proxy, was the highest in the center of the nucleus (Girelli et al., 2020). It has been shown previously that the deterministic parameter for radial positioning of chromatin in the nucleus is regional gene density (Kupper et al., 2007). Therefore, these results support our hypothesis that DMSO- and APH-induced DSBs are enriched in gene-dense regions with high level of transcription. We next investigated conservation of sequence motifs within the DSB regions in an effort to identify additional evidence for transcription-dependent mechanism of DSB formation.

### Sequence motif discovery in replication stress-induced DSBs revealed binding sites for transcription factors implicated in maintaining 3D chromatin architecture

To specifically delineate the effect of DMSO-mediated transcription induction and APH-mediated replication stress on DSB formation, we analyzed the DMSO- and APH-specific DSBs, respectively. Motif search using AME (Analysis of Motif Enrichment, (McLeay and Bailey 2010)) identified a dearth of transcription factor (TF) binding sites in both groups. The top ten motifs enriched in DMSO only DSBs are binding sites for *ZNF582, SMARCA5, ZNF770, STAT5B, ZNF121, PAX5, STAT5A, PRDM6, TAF1,* and *ZNF418* (*p* < 5.59e-42). The top ten motifs enriched in APH only DSBs are binding sites for *ETS2, EGR2, EGR1, NFATC1, ETV5, LEF1, TBX21, ZNF341, BCL11A,* and *ZNF121* (*p* < 5.16e-124). Importantly, CTCF binding motif was also found as enriched in APH only DSBs (*p* = 5.74e-5). Many of the proteins above (e.g., PRDM histone methyltransferase, the SMARCA subgroup of genes belonging to the SWI1/SNF1 chromatin remodelers, etc.) have been implicated in chromatin modifications and remodeling. Others have been implicated in the maintenance of 3D chromatin architecture. For instance, ZNF770 and ZNF121 are significantly enriched at “insulator loops” mediated by CTCF to protect genes from emanating potentially harmful signals (Trieu et al., 2020). PAX5, a transcription factor essential for B-cell identity and function, changes 3D chromatin architecture (van Schoonhoven et al., 2020). Yet other TFs themselves are subjected to regulation for expression by chromatin architecture. For instance, CTCF has been shown to regulate and promote expression of EGR1 and EGR2 by establishing chromatin interaction loops between enhancer and promoter regulatory elements (Sekiya et al., 2019; Wang et al., 2020). We also used STREME (Bailey 2021) to discover new motifs in these groups, followed by identification of similar known motifs by Tomtom (Gupta et al., 2007) (Table 1). The results confirmed that DSB regions in both groups were enriched for sites for TF binding, thus supporting the notion that DSBs are transcription-dependent.

**TABLE 1:** Select discovered motifs in DMSO unique DSBs and APH unique DSBs.

Motif (P < 1e-08)	Logo	*p*-Value	Sites	Similar Motifs (*p* < 0.01)
DMSO-specific DSBs				
GCCTCAGCCTCCCRA	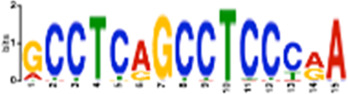	2.3e-10	318 (14%)	ZN770, IKZF1, ZN281, SALL4, CRX, KLF4, SMAD3, TBX21
AATCTGCAAGTGGAT	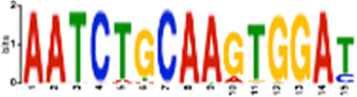	3.7e-09	336 (14.8%)	PO2F2, ELF1, ELF3, ELF2, EHF, PRDM1, IRF2, CLOCK, IRF1, ETS1, SPI1, ELF5, ETV5, ERG
CACTGCACTCCAGCC	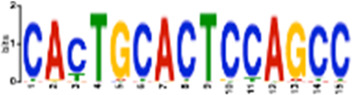	3.7e-09	278 (12.2%)	ZSC31, TEAD1, SMCA5, NR2C1, NKX21, NKX25, ZN502
GGTTCAACTCTGTGA	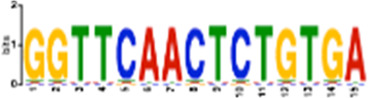	7.4e-09	315 (13.9%)	ZN768, ZF331, VDR, ZKSC1
AACTGCTCWATCAA	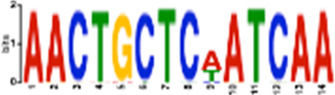	7.4e-09	314 (13.8%)	NF2L2, MAF, MAFG, MAFF, MAFB, CEBPD, PDX1, MAFK
AAACTTCTTTGTGAT	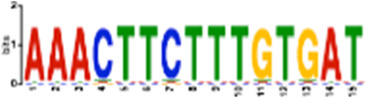	7.4e-09	312 (13.7%)	NF2L2, ZN680, SMCA1
APH-specific DSBs				
CCTCAGCCTCCCRA	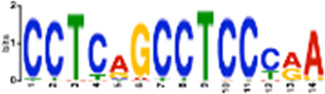	4.4e-16	499 (14.6%)	ZN770, IKZF1, ZN281, CRX, SALL4, ETS2, TBX21, WT1, SMAD3, SRBP2
CCAGCCTGGGCRACA	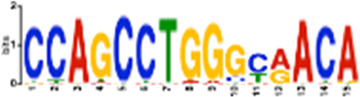	4.5e-14	539 (15.8%)	PAX5, SUH, ZN121, RFX2
GCTGGGATTACAGGC	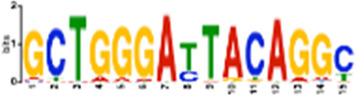	2.3e-13	483 (14.1%)	ZN264, GFI1, GFI1B
CAGTGAGCYGAG	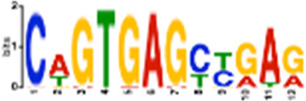	1.3e-12	469 (13.7%)	RARG, SRBP1, NR1H3, NFIC, ZN331, SRBP2, BRAC, RXRB
GAATYGCTTGAAC	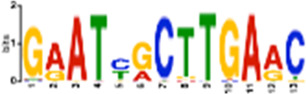	1.2e-10	369 (10.8%)	ZN140, PBX2, ZN490, ZN329
TCTACWAAAA	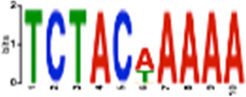	1.4e-10	386 (11.3%)	TBP, MEF2C, MEF2A, ANDR, ZN490
GARACCCCRTC	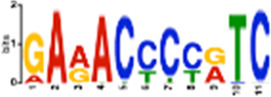	4.0e-09	327 (9.6%)	RUNX1, ZBT7A

### Identifying classifier(s) for DMSO- and APH-induced DSBs using machine learning

**FIGURE 7:**
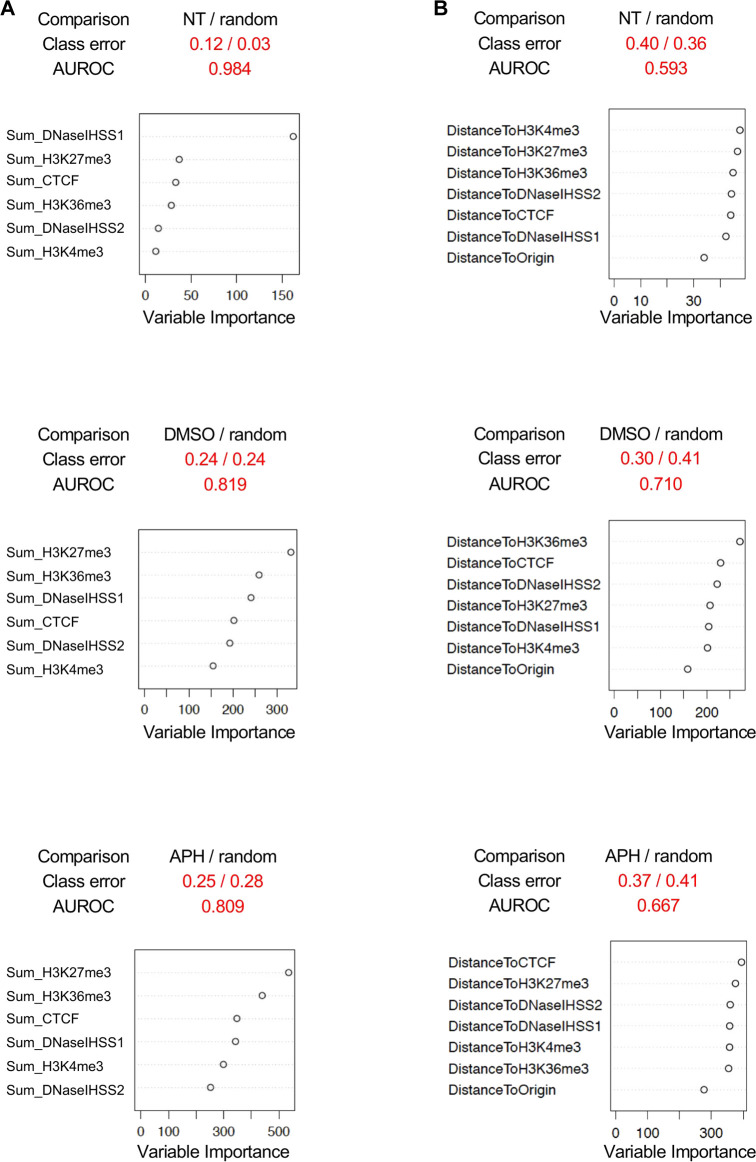
Random forest analysis. **(A)** Comparisons between DSBs specific to each treatment, NT, DMSO, and APH, and randomly sampled DSBs from the genome using feature signals over DSBs. **(B)** Comparisons between DSBs specific to each treatment, NT, DMSO, and APH, and randomly sampled DSBs from the genome using feature distance to DSBs. AUROC, area under the receiver operating characteristic curve.

As a final endeavor, we asked if we could predict DSBs using these genomic and epigenomic features (henceforth “features” for simplicity). Motivated by Mourad et al.'s use of a machine learning method, Random Forest, to distinguish spontaneous DSBs from randomly sampled genomic regions, we developed a similar pipeline to validate features for replication stress-induced DSBs (Breiman 2001; Mourad et al., 2018). To best delineate the classification, we focused on the DSBs unique to each treatment rather than the entire cohort of DSBs in each treatment (Figure 1A). We then randomly sampled the hg19 genome for non-DSB regions with the same chromosome and length distributions as those DSBs in each treatment group for comparisons, *i.e.*, non-NT/NT, non-DMSO/DMSO, and non-APH/APH. We assessed the variable importance of distance to DSBs by each feature. Note, we also analyzed the variable importance of feature signals over DSBs, computed as the sum or mean of signals across the DSBs from downloaded ChIP-seq bigWig data of GM06990 from the ENCODE project (Supplementary Figure S4). We then trained Random Forest to recognize the “real” DSBs, with reasonable accuracy (AUROC >0.8, class errors <0.3, *i.e.*, >70% accuracy) using feature signals over DSBs (Figure 7), and with less accuracy using distance to features (Figure 7). By far the best predictor for NT, or spontaneous DSBs was DNaseI HSS (experiment 1) (Figure 7A). This result agrees with the findings by Mourad et al. and suggests that spontaneous DSBs occur at regions with high chromatin accessibility. The best predictor for both DMSO- and APH-specific DSBs was H3K27me3, followed by H3K36me3 (Figure 7B), indicating that both transcription induction and repression are correlated with DSBs. However, it is unclear whether the repressive histone marker is a consequence rather than a cause for the DSB. Moreover, high feature density might bias the results using feature signal over DSBs. Therefore, we then analyzed the results based on feature distance from DSB. In comparison, the class errors for feature distance were moderately higher than feature signal (Figures 7A,B). The best predictors for DSBs in NT, DMSO, and APH samples are H3K4me3, H3K36me3, and CTCF, respectively (Figure 7B). Among these, the role of active transcription in driving DMSO-specific DSBs was most apparent. These results corroborated our conclusions insofar as active transcription, as well as TAD domain boundaries, played an important part in induction of DSB formation. In summary, the inclusion of the Random Forest model in this study proved to be a promising tool in determining specific correlations between genomic and epigenomic features within the spontaneous and replication stress-induced DSBs.

## Discussion

In this report, we detail the computational analysis of spontaneous and replication stress-induced DSBs in GM06990 lymphoblastoids, which were recently mapped by Break-seq (Chakraborty et al., 2020). We focused on understanding the relationship between DSB formation and epigenomic and genomic signatures. Importantly, by parsing the DSBs to those specifically induced by DMSO, or by APH, it allowed us to dissect the effect of the replication inhibitor from its solvent independently.

The first key finding was that DSBs were closely associated with active transcription histone marker H3K36me3 and TAD boundaries. Specifically, spontaneous DSBs are associated with CTCF binding sites. In contrast, we observed a correlation between DMSO-induced DSBs and markers associated with active transcription, including TSSs, H3K36me3 and CTCF binding sites. Upon APH treatment, there was a dampening of the transcriptional response as evidenced by a decrease in the levels of all markers above, as well as a decrease of DSBs in genic regions compared to DMSO treatment. Yet, the APH-induced DSBs showed strong association with H3K36me3, suggesting that despite an overall dampening of transcription by APH DSBs nevertheless took place at actively transcribing regions of the genome.

The second key finding related to the first one was that APH-induced DSBs in our study as well as the BLESS study (Crosetto et al., 2013; Chakraborty et al., 2020) were not enriched in large genes (>300 kb in size) and did not correlate with the CFS core sequences. Instead, we observed a tendency of the APH-induced DSBs to demarcate the boundaries of CFSs or the CFS core sequences. We suggest that DSBs occur at TAD boundaries marked by CTCF. Because the density of CTCF binding at large genes is relatively lower than that at smaller genes, DSB frequency is accordingly lower at large genes. However, we note that our Break-seq data were generated with relatively low level of APH (0.03 and 0.3 µM), while the BLESS study used 0.4 µM APH. Therefore, it is possible that the DSBs within the CFS cores might be more readily detected at higher level of APH.

**FIGURE 8:**
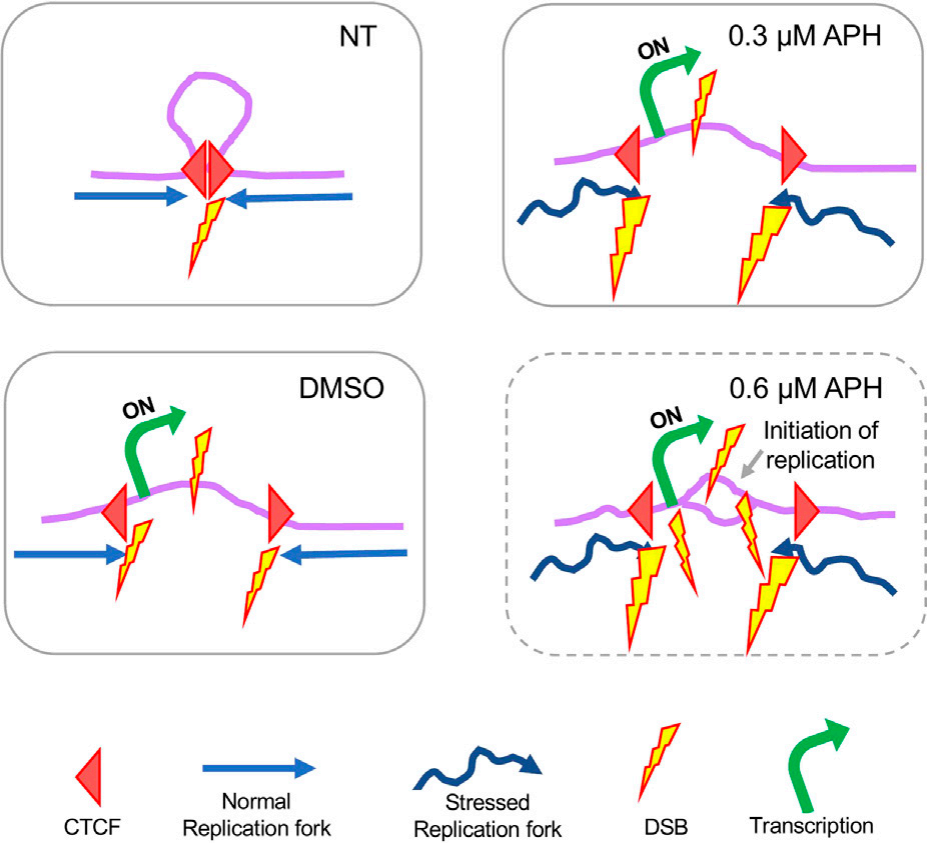
Proposed model of DSB formation at large genes organized by chromosome looping through CTCF binding. For details please see discussion in the main text.

Overall, these findings led us to hypothesize that DSBs were the result of CTCF-mediated chromosome remodeling due to transcription. We propose the following model to explain spontaneous and drug-induced DSB formation, specifically at the large genes where CFSs tend to reside (Figure 8). In untreated cells, DSBs occur at those CTCF binding sites involved in chromosome looping as replication forks originated from either side of the loop progress towards them. Thus, these DSBs form a bifurcated distribution over CTCF binding sites, as observed in Figure 5. Upon DMSO treatment, transcription induction causes the chromosome loop to disassemble, exposing the CTCF binding sites to replication forks on both sides and reducing the appearance of a bifurcated distribution pattern of DSBs over these sites. Meanwhile, active transcription within the chromosome loop also causes DSBs, either independently or when encountering approaching replication forks. In 0.3 µM APH, transcription remains active at large genes despite a global dampening of transcription. Additionally, unstable replication forks increase the probability of DSBs at CTCF binding sites (denoted by larger DSB icons) and/or active transcription sites. Finally, replication timing study indicated that in cells treated with 0.6 µM APH there were localized initiation events within the CFS core regions, suggesting that DSBs were produced by unscheduled replication termination with forks initiated from outside the CFS region. This observation provided an extended explanation for chromosome breakage at the CFS core regions. This model is consistent with a recent study presenting strong evidence that initiation of origin activation involves transcription-induced reorganization of the TAD demarcated by CTCF binding, which presents select origins within the TAD to move to the periphery for efficient activation (Li et al., 2021).

In summary, our study provided a comprehensive overview of genomic and epigenomic features associated with replication stress-induced DSBs. It also laid out a framework for future studies to expand DSB mapping to more well-chosen cell lines and with simultaneous queries for active origins of replication and epigenomic features. Such an experimental design will promise to deliver important insights into the mechanisms of replication stress-induced DSB formation.

## Data Availability

The original contributions presented in the study are included in the article/Supplementary Materials, further inquiries can be directed to the corresponding author.
