# Supplementary material for: Epigenomic signatures associated with spontaneous and replication stress-induced DNA double strand breaks
Source: Front Genet. 2022 Nov 24;13:907547. doi: 10.3389/fgene.2022.907547 (PMC9730818; doi:10.3389/fgene.2022.907547)
Supplement: Supplementary file 1 [file DataSheet1.PDF]

Supplementary Material

Supplementary Data

DataFileS1.xlsx

Sheet 1: "ntDSB.CTCF.H3K36me3.origin.overlap.bed"

Sheet 2: "dmsoDSB.CTCF.H3K36me3.origin.overlap.bed"

Sheet 3: " aphDSB.CTCF.H3K36me3.origin.overlap.bed"

Supplementary Figure Legends

Supplementary Figure 1. Chromatin features used for analysis with respect to DSBs. (A) Total number of genomic locations in each chromatin feature. Data for all features except for Origin were extracted from the ENCODE project (Methods). Data for origins were extracted from Mesner et al. 2013, Genome Res. **(B)** Stacked column plots showing genomic distribution of each feature group relative to genes.

Supplementary Figure 2. Drug-induced DSBs are enriched at locations where CTCF binding sites, H3K36me3 and TSS converge. (**A**) Aggregated plots of DSBs around ALL TSS and H3K36me3 genome-wide. (**B-D**) Aggregated plots of chromatin makers as indicated around the nearest (left column) or all (right column) markers as indicated. Binning of all features are identical as in Figure 4.

Supplementary Figure 3. Aggregated plots of origins around the nearest chromatin markers as indicated. The number of DSBs in each of the 50 bins across a 20,000 bp window centered on the given chromatin marker are scored and plotted on the Y-axis against the relative distance to the center of the chromatin marker (X-axis).

Supplementary Figure 4. Box plots of average signals of epigenomic features over DSB regions. Shown are complete sets of DSBs ("NT", "DMSO", and "APH") as well as subsets of DSBs ("NT-specific", "DMSO-specific", and "APH-specific") as derived from Fig. 1A.

Figure S1

A

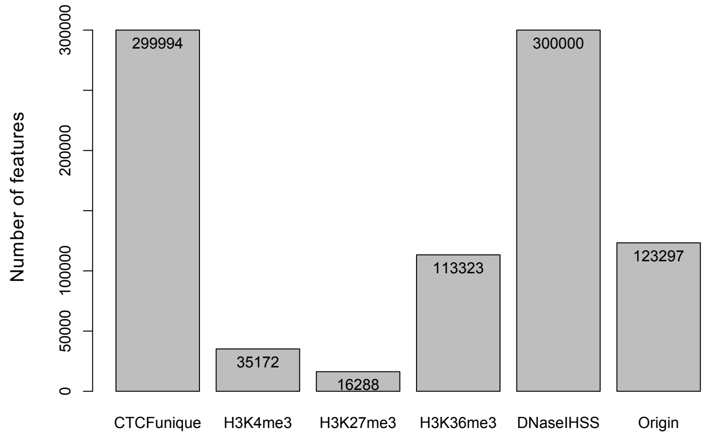

Number of features
0
50000
100000
200000
300000
299994
35172
16288
113323
300000
123297
CTCFunique
H3K4me3
H3K27me3
H3K36me3
DNaseIHSS
Origin

B

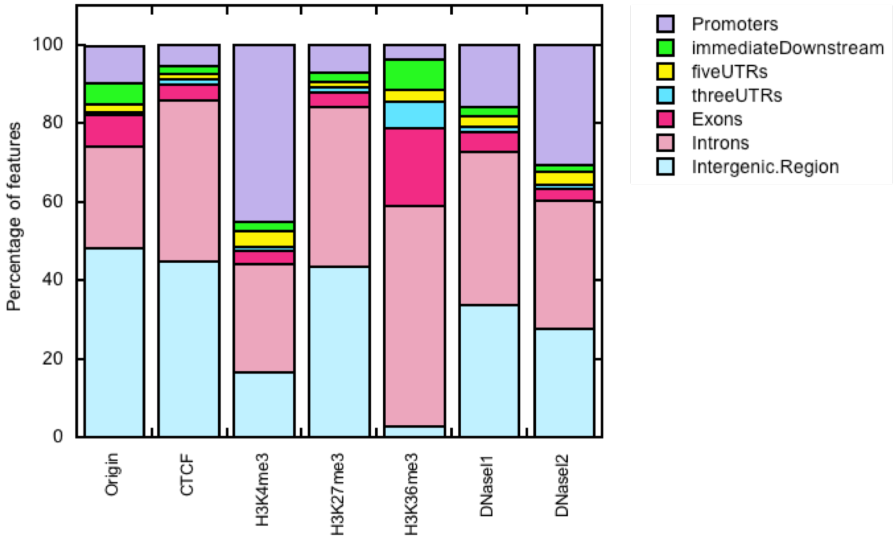

Percentage of features
0
20
40
60
80
100
Origin
CTCF
H3K4me3
H3K27me3
H3K36me3
DNaseI1
DNaseI2
Promoters
immediateDownstream
fiveUTRs
threeUTRs
Exons
Introns
Intergenic.Region

Figure S2

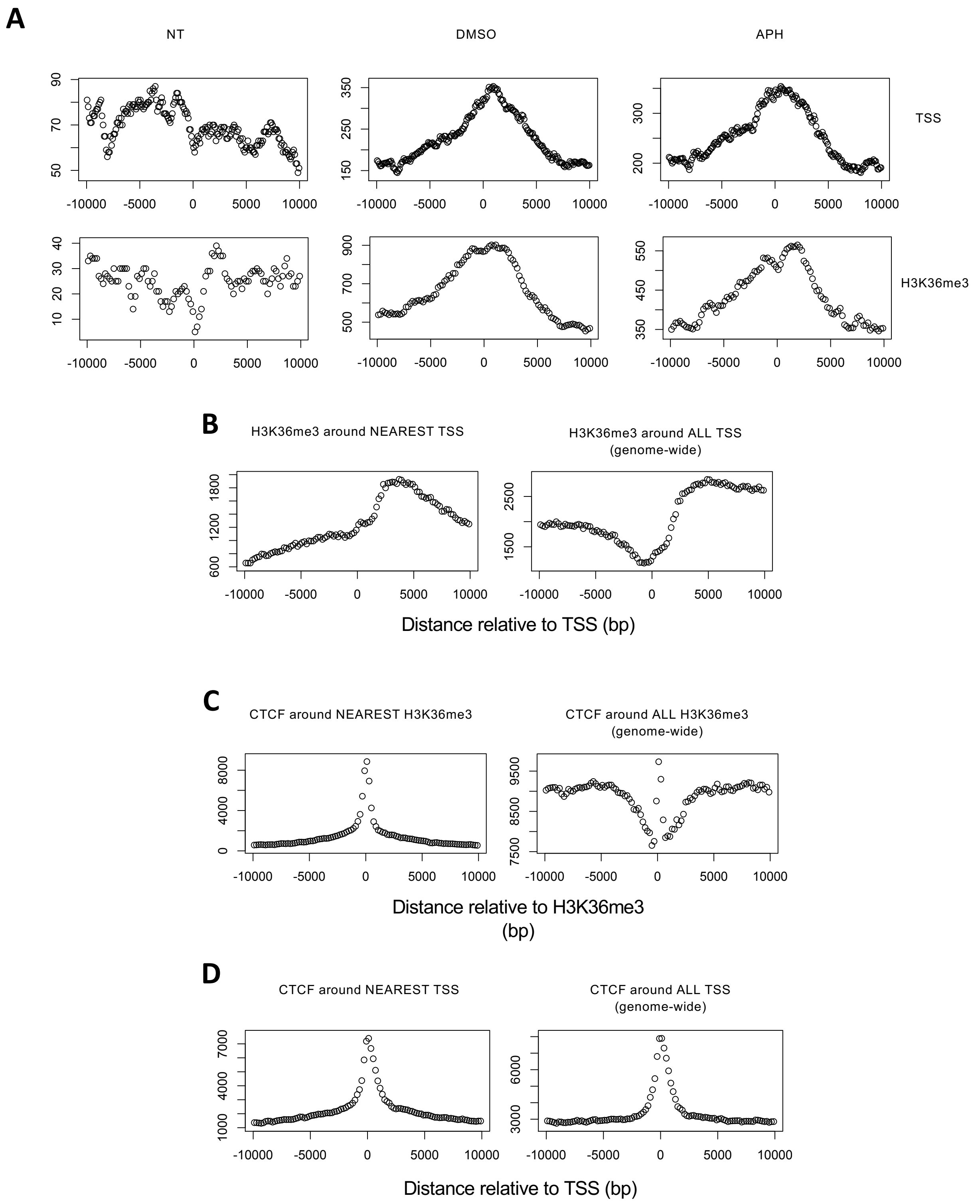

A
NT
DMSO
APH
TSS
H3K36me3
B
H3K36me3 around NEAREST TSS
H3K36me3 around ALL TSS (genome-wide)
Distance relative to TSS (bp)
C
CTCF around NEAREST H3K36me3
CTCF around ALL H3K36me3 (genome-wide)
Distance relative to H3K36me3 (bp)
D
CTCF around NEAREST TSS
CTCF around ALL TSS (genome-wide)
Distance relative to TSS (bp)

Figure S3

A

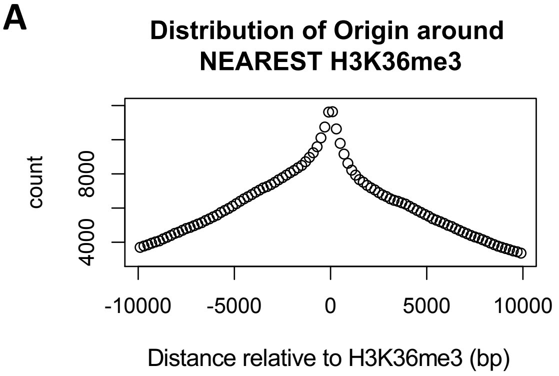

B

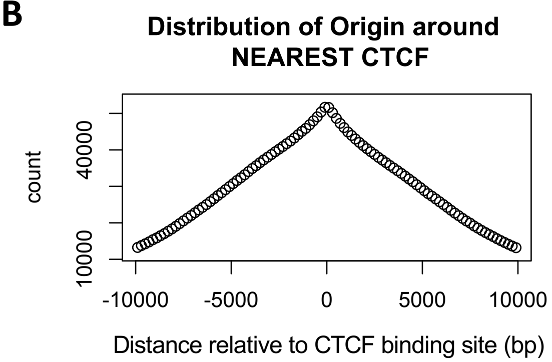

C

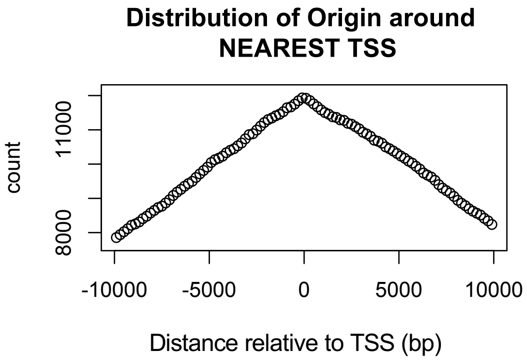

Figure S4

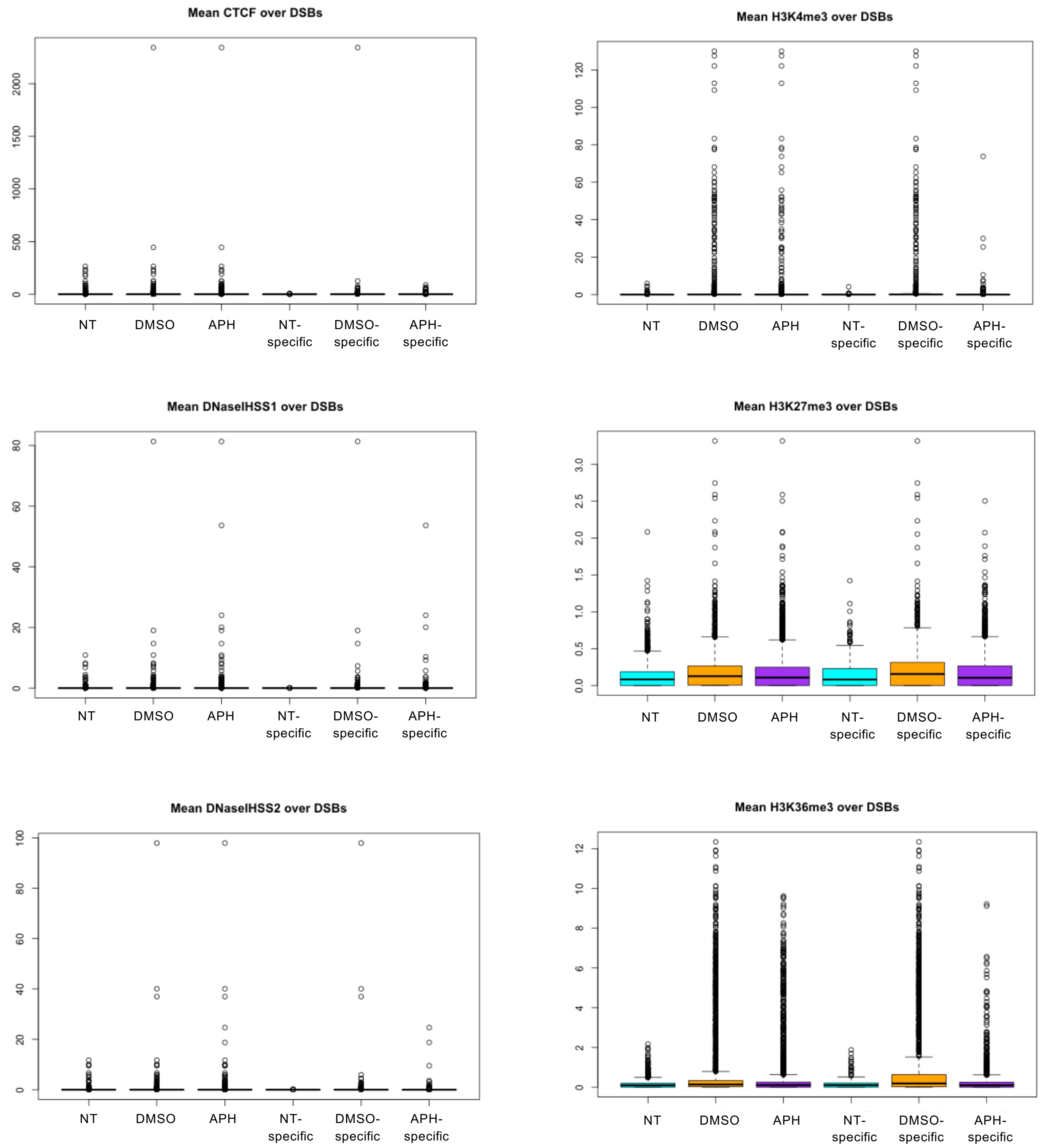
Mean CTCF over DSBs
0
500
1000
1500
2000
NT
DMSO
APH
NT-specific
DMSO-specific
APH-specific
Mean H3K4me3 over DSBs
0
20
40
60
80
100
120
NT
DMSO
APH
NT-specific
DMSO-specific
APH-specific
Mean DNaseIHSS1 over DSBs
0
20
40
60
80
NT
DMSO
APH
NT-specific
DMSO-specific
APH-specific
Mean H3K27me3 over DSBs
0.0
0.5
1.0
1.5
2.0
2.5
3.0
NT
DMSO
APH
NT-specific
DMSO-specific
APH-specific
Mean DNaseIHSS2 over DSBs
0
20
40
60
80
100
NT
DMSO
APH
NT-specific
DMSO-specific
APH-specific
Mean H3K36me3 over DSBs
0
2
4
6
8
10
12
NT
DMSO
APH
NT-specific
DMSO-specific
APH-specific
